# Maternal Oral Health and Global Oral Health: Policy Perspective

**DOI:** 10.1093/eurpub/ckac129.261

**Published:** 2022-10-25

**Authors:** K Ramphoma, H Lee

**Affiliations:** 1 Department of Community Oral Health, University of the Western Cape, Cape Town, South Africa; 2 Oral Health Workgroup, WFPHA, Geneva, Switzerland

## Abstract

**Background and problems:**

The findings of the last South African National Children Oral Health Survey conducted between 1999 and 2002 indicated a national prevalence of dental caries at 60% among 6-year-olds, with over 80% of untreated caries. Various studies have shown that one of the successful strategies in preventing dental caries is intervention during pregnancy by providing prenatal oral health education.

**Importance and opportunities:**

To successfully mitigate the challenge of ECC, South Africa needs a holistic approach strategy that will integrate oral health services into existing general health programmes, especially into antenatal care (ANC). South Africa National Oral Health Policy and Strategy states that “Integrate oral health strategy elements and strategies into programmes and policies of all sectors that impact community health like maternal and women's health.” All government health care facilities offer free essential ANC services, identifying risk factors relating to poor maternal and birth outcomes. This existing infrastructure can be utilized to deliver oral health education and care for expecting mothers as essential ANC services.

**Solution:**

The Public Oral Health Forum, a non-profit organization in South Africa, seeks to develop maternal and oral health policies that can be aligned with its research and policy development strategy. It proposes maternal oral health policy and strategies, which could be applicable in the South African context: Education of pregnant women, prenatal providers, and dental and medical students about perinatal and infant oral health, integration of oral health in primary health care services, interprofessional workforce development and research to improve oral health and access to dental care among pregnant women and new mothers. This presentation will demonstrate how such maternal oral health policy and strategy are developed, aligning with existing policy and infrastructure in South Africa.

